# The shades of grey in adipose tissue reprogramming

**DOI:** 10.1042/BSR20212358

**Published:** 2022-03-08

**Authors:** Yue Qi, Xiaoyan Hui

**Affiliations:** School of Biomedical Sciences, The Chinese University of Hong Kong, Hong Kong, SAR

**Keywords:** adipose tissue, cellular senescence, chronic inflammation, circadian rhythm, COVID-19, obesity

## Abstract

The adipose tissue (AT) has a major role in contributing to obesity-related pathologies through regulating systemic immunometabolism. The pathogenicity of the AT is underpinned by its remarkable plasticity to be reprogrammed during obesity, in the perspectives of tissue morphology, extracellular matrix (ECM) composition, angiogenesis, immunometabolic homoeostasis and circadian rhythmicity. Dysregulation in these features escalates the pathogenesis conferred by this endometabolic organ. Intriguingly, the potential to be reprogrammed appears to be an Achilles’ heel of the obese AT that can be targeted for the management of obesity and its associated comorbidities. Here, we provide an overview of the reprogramming processes of white AT (WAT), with a focus on their dynamics and pleiotropic actions over local and systemic homoeostases, followed by a discussion of potential strategies favouring therapeutic reprogramming. The potential involvement of AT remodelling in the pathogenesis of COVID-19 is also discussed.

## Introduction

Obesity, now a global epidemic, is a multifaceted metabolic disease characterised by excessive adiposity and adipose tissue (AT) dysfunction [1,2]. It is estimated that more than 1.9 billion adults worldwide are currently living with obesity or are overweight [3]. Obesity is a significant driver of non-communicable diseases and is consistently associated with increased all-cause mortality across different ethnicities [4,5]. On the surface, obesity is a manifestation of chronic overnutrition, especially the overconsumption of ultraprocessed foods, which is compounded by sedentary lifestyles and misaligned diurnal eating patterns/windows [6–8]. However, excessive adiposity *per se*, even in the absence of other full-fledged pathology, has profound implications on cardiometabolic risk over the course of life span [9,10].

The white AT (WAT), due to its ability in buffering excessive energy and regulating systemic metabolic health, is closely associated with the pathogenesis of obesity-related morbidities and is at the heart of obesity research. In addition to adipocytes, the AT is a heterogeneous organ that harbours diverse cell populations, including immune cells, stromal/stem cells, fibroblasts, endothelial cells (ECs), and smooth muscle cells, which orchestrate AT and systemic homoeostasis [11,12]. The role of WAT goes beyond an energy sink that handles energy surplus. In fact, the crux of the matter in obesity is the pathologic reprogramming of WAT, a multifaceted process that involves the changes in intra- and extra-cellular signalling, cell functionality and identity, cell–cell communications, and extracellular composition. Specifically, the plasticity of WAT allows it to be functionally reprogrammed in response to repeated episodes of feasting through the recruitment of immune cells, secretion of pro-inflammatory adipokines, and remodelling of extracellular matrix (ECM). As a consequence of the maladaptive reprogramming, the WAT develops an inflammatory and fibrotic phenotype associated with cellular senescence, escalating systemic low-grade inflammation and metabolic impairments [13–15].

**Figure 1:**
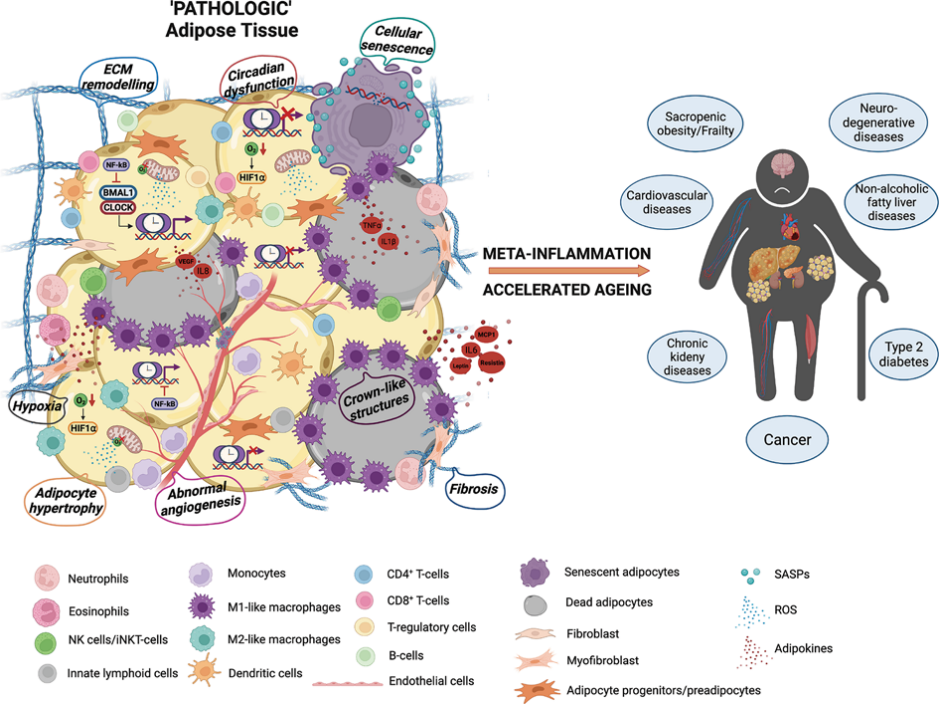
Pathologic reprogramming of WAT as the major culprit of obesity-associated diseases As a highly plastic endocrine organ, the AT undergoes extensive remodelling which involves hyperplasia/hypertrophy, fibrosis, angiogenesis and hypoxic response, chronic inflammation, cellular senescence and clock dysfunction. These abnormalities intertwine and escalate meta-inflammation and premature ageing, which ultimately manifest as obesity-related morbidities.

On the other side of the coin, AT can also engage in thermogenesis or energy burning through uncoupling the oxidative phosphorylation and ATP production, where protons are shuttled through uncoupling protein-1 (UCP1) to generate heat [16]. These thermogenic AT depots, namely the brown or beige AT, are recruited and activated during cold exposure or pharmaceutical induction of adrenergic receptors [17,18]. An appreciated amount of the classical interscapular brown AT, characterised by homogenous multilocular adipocytes and constitutively high expression of UCP1, is present in rodents and human newborns and acts as a defensive mechanism for cold stress [19]. Whereas in the human adults, the interscapular depot is diminished, but alternative thermogenic AT depots consisting of both brown and beige adipocytes [20] are identified around the cervical, supraclavicular, paraspinal and suprarenal regions using the combined position positron emission tomography and computed tomography (PET-CT) [21–23]. Although it remains debatable whether brown/beige AT contributes to a meaningful amount of energy expenditure, hence facilitates weight loss [24], the fact that these depots benefit cardiometabolic health beyond thermogenesis and regulate systemic metabolism [25–29] grants the therapeutic potential of reprogramming the AT into an energy-burning ‘furnace’, which has been eloquently reviewed elsewhere [30]. In this review, we will outline the various facets behind the pathological reprogramming of AT, particularly of the WAT (Figure 1), whose multifarious and dynamic roles during obesity development and progression are discussed.

## Physical reprogramming of AT

The transition from a lean to an obese phenotype is accompanied by the dimensional expansion of AT, where both hypertrophy (increase in cell size) and hyperplasia (increase in cell numbers) are employed in a depot-specific manner to accommodate the overwhelming energy reserve. Insights regarding the relative contribution of hypertrophy and hyperplasia have been offered by the AdipoChaser mice, in which a high-fat diet (HFD) triggers hypertrophy initially but preferentially induces *de novo* adipogenesis of the visceral depot in the long term [31]. In rodents, the shift towards hyperplasia likely occurs once the enlarged adipocytes exceed certain threshold volumes. But the story becomes more complicated in humans. It has been suggested that the numbers of adipocytes are similar between normal-weight individuals and patients with obesity, whereas the size of adipocytes becomes distinguishably larger in obesity [32]. Spalding et al. reported that the number of one’s adipocytes is established during childhood and adolescence, which remains stable through a constant rate of turnover and is resistant to change upon weight fluctuations in adulthood [33]. However, people with obesity are also found to reach a higher level of adipocyte number threshold earlier in their life [33]. Interestingly, an subsequent overfeeding trial demonstrated that the abdominal subcutaneous AT (SAT) and mid-thigh SAT respond to a nutritional challenge by hypertrophy and hyperplasia respectively [34]. Nevertheless, hypertrophy in both visceral AT (VAT) and SAT has been suggested as a significant risk factor for cardiometabolic derangements across different ethnicities [35,36]. In fact, hypertrophy, especially in the visceral depot, is recognised as a maladaptive response of WAT, which is accompanied by down-regulation of adipogenesis and insulin sensitivity and but heightened cellular stress and inflammatory profile [37,38]. However, the morphological reprogramming of AT may also exert important physiological and adaptive effects. Smaller adipocytes, compared with their larger counterparts, have been positively associated with inflammatory markers and obesity comorbidities [39,40], supporting that size of adipocytes *per se* does not contribute to AT dysfunction [40,41]. Instead, the reduced AT expandability and buffering capacity appear to underlie pathological reprogramming [42]. Collectively, both hypertrophy and hyperplasia could mediate human obesity, but the former mode of expansion entails deleterious consequences. What are the consequences of going bigger?

## Remodelling of ECM

The ability of WAT to enlarge is facilitated by its ECM, which consists of proteins, polysaccharides, and proteoglycans and offers a flexible mechanical and structural scaffold for AT and a biochemical framework for cell–cell communication [43]. Functional and epigenetic reprogramming of the ECM is crucial for maintaining the microenvironment homoeostasis of AT, hence influencing systemic metabolism and inflammatory milieu [44]. In contrast, AT fibrosis, due to an abnormal build-up of ECM constituents, down-regulates the plasticity of adipocytes and propagates inflammatory signals [45]. However, the link between AT fibrosis and clinical manifestations in humans is yet well-defined and is influenced by AT depot and population ethnicity. Fibrosis in VAT, the depot that is more closely related to obesity complications due to proximity to internal organs [46], seems to be an adaptive response to limit its expansion while promoting hyperplasia and preventing tissue dysfunction. Studies comparing the degree of fibrosis in human AT have suggested an inverse relationship between VAT fibrosis and symptomatology and progression of type 2 diabetes (T2D) and non-alcoholic fatty liver disease, although these might have been confounded by medication use [47,48]. On the other hand, fibrosis in SAT is positively associated with pre-diabetes and insulin resistance (IR), with the relationship being more prominent in Chinese ethnic background [49,50]. In addition, SAT fibrosis has been negatively associated with weight loss after bariatric surgery [51]. Notably, the other study found that the physical stiffness of AT, but not the surrogate marker of fibrosis (collagen), is associated with patients’ clinical presentation [52]. Therefore, more comprehensive methods that include both biochemical and physical measures shall be adopted when assessing AT fibrosis in future.

Fibrosis of WAT is intricately linked to an imbalance between the degradation and synthesis of ECM components. Among these components, collagen proteins, which are primarily secreted from adipocytes, have been extensively investigated. Up-regulation of collagen expression, specifically collagen VI, has been observed in both human and murine obesity and is positively correlated with pathogenic ECM remodelling [53]. In particular, the visceral adipocytes develop a fibroblast-like transcriptome characterised by overt expressions of collagen VI, fibronectin and pro-fibrotic cytokine transforming growth factor-β (*Tgfb*) in response to HFD [54]. The fibrotic signal in AT is also amplified by suppressing the adipogenic potential, where adipocyte progenitors are rewired into myofibroblast cell fates [55]. Collagen VI knockout mice are protected from genetic and diet-associated weight gain and ectopic fat deposition, accompanied by better insulin sensitivity despite having larger adipocyte size. Similarly, adipocyte-specific overexpression of endotrophin, a cleavage product of collagen VI, induces ECM fibrosis and inflammatory responses, while neutralisation of endotrophin attenuates AT inflammation and confers protection against HFD-induced IR and dyslipidaemia [56]. Mechanistically, deletion of collagen VI reduces the rigidity of ECM through regulating lumican, decorin and elastin, and thereby protects the adipocytes from mechanical stress and necrotic death during expansion, which would otherwise contribute to the formation of ‘crown-like’ structures (CLSs) and an inflammatory signature [53]. Furthermore, the ECM has been reported to reprogramme adipocyte metabolism and contribute to the phenotypic differences between visceral and subcutaneous depots. Culturing visceral adipocytes in subcutaneous ECM rescues HFD-induced IR and promotes adipogenesis, whereas visceral ECM dampens glucose uptake and expression of adipogenic genes in subcutaneous adipocytes [57]. Notably, in brown AT, reduced expressions of *Tgfb* and its downstream collagen genes are associated with an impairment in brown AT thermogenesis [58]. These findings indicate that the AT resident cells and ECM components act synergistically to facilitate the physiological and pathological AT reprogramming.

## Angiogenesis and hypoxia

What prompts the bigger AT to go harder at the same time? Current thinking suggests that dysregulated angiogenesis and associated hypoxia due to a crowded AT niche are the main initiating factors of fibrosis. The AT is among one of the most vascularised tissues with each adipocyte surrounded by a capillary network [59], much like a ‘mesh squishy ball’. Angiogenesis is required to meet the oxygen demand, to deliver the nutrients and hormones, and to remove waste products of the tissue. Recruitment of new blood vessels is initiated by up-regulation of angiogenic factors, including leptin, vascular endothelial growth factor (VEGF) and TGFβ, released from adipocytes, stromal cells, and immune cells. Note that as much as 50% of the AT secretome represents angiogenic modulators [60]. SAT taken from young people with obesity but are otherwise healthy has significantly altered expressions of genes involved in angiogenesis and ECM remodelling, rather than those implicated in inflammatory processes [61]. This implies that the abnormal angiogenesis, together with ECM remodelling, precedes inflammatory cascades in AT. It is likely that angiogenesis is an attempt made by the AT during acute expansion to appropriately reprogramme and accommodate energy influx, while over time the failure to do so subsequently leads to tissue dysfunction. In support of this view, gene expression of VEGF is up-regulated upon 3 days of HFD, while impaired AT capilarisation and abnormal tissue levels of VEGF are associated with human chronic obesity [62], especially among those with IR [63,64]. Administration of anti-angiogenic agents, such as TNP-470, before the development of diet-induced obesity (DIO), has been shown to be anti-obesity [65], implying that initiation of angiogenesis in the lean fat may serve as an essential prerequisite for obesogenesis upon overnutrition. However, the antidiabetic medication thiazolidinedione exerts its anti-inflammatory benefit through promoting capillary density, which helps support the healthy expansion of adipocytes, alleviating hypoxia and inflammation [66]. Likewise, overexpression of VEGF in white and brown AT exhibits increased vascular density of AT and attenuated hypoxia, accompanied with a relative enrichment of anti-inflammatory M2 macrophages, followed by amelioration of DIO and IR [67]. Furthermore, activation of VEGFB/VEGF receptor (VEGFR)-1 signalling confers protection against obesity-induced metabolic complications and AT inflammation through restoring insulin sensitivity and optimal vasculature in VAT, as well as inducing expression of *Ucp1* in SAT, implying the role of angiogenesis in supporting beiging [68]. Nevertheless, higher vascular density and an increased expression of VEGFR2 have been observed in VAT compared with the subcutaneous depot, which is coupled with enhanced AT inflammation and hypoxia in participants with obesity, suggesting that angiogenesis mediated by VEGFA/VEGFR2 signalling is associated with a pathogenic visceral microenvironment [69]. Further investigation is required to resolve the mechanisms contributing to the pathologic angiogenesis in obesity as well as the influences of different AT niche and metabolic signals in mediating the physiological roles of angiogenic responses.

In contrast with the oncogenic vascularisation where oxygen insufficiency triggers angiogenesis [70], hypoxia of AT fails to stimulate an angiogenic response, but instead, elicits a reprogramming process mainly mediated by hypoxia-inducible factor-1α (HIF1α) [71]. This is evidenced by the observation that the HIF1α level is significantly increased in VAT after 4 days of HFD, followed by an up-regulation of fibrotic proteins [71]. Furthermore, overexpressing HIF1α induces AT dysfunction, which is reflected by systemic glucose intolerance and an increased immune cell filtration and enhanced fibrosis of AT primarily through up-regulating lysyl oxidase (LOX). The deleterious role of hypoxia is further demonstrated that inhibition of HIF1α, either by treatment with PX-478 or AT-specific deletion, alleviates AT fibrosis and dysfunction and rescues metabolic deterioration and HFD-induced pathologies [72]. The influence of hypoxia on AT fibrotic potential is also detailed by a recent study using an *in vitro* three-dimensional AT model in which hypoxia leads to a fibrillar assembly of fibronectin, which is a major trigger of aberrant deposition of ECM [73], as well as imposing mechanical stress to adipocytes through the formation of actin stress fibres that amplifies the synthesis of the fibrillar matrix [74]. Interestingly, increased energy expenditure and up-regulated expressions of *Ucp1* and *Pgc1a* in the SAT are also observed in the PX-478-treated mice, demonstrating the AT beiging is mobilised upon inhibiting the AT hypoxia response [75]. Further investigation is needed to unravel whether beiging is secondary to or a direct target of HIF1α inhibition.

Abnormal hypoxia signalling observed in rodents is also mirrored by human AT during obesity, where a reduced oxygen partial pressure (PO_2_) and/or overexpression of HIF1α in SAT, specifically in AT-derived macrophages (ATMs), have been identified in individuals with metabolically unhealthy obesity compared with lean individuals, although the clinical significance of AT hypoxia is debatable [76–79]. In contrast, Goossens et al. reported that PO_2_ is paradoxically high in the obese AT [80]. However, the AT PO_2_ observed in individuals with obesity ranged from 20 to 90 mmHg and overlapped with that of lean subjects. Given that there is no defined cutoff for diagnosing AT hypoxia in humans, it can be argued that higher AT PO_2_ in participants with obesity is not entirely attributed to physiologically relevant hyperoxia, but instead, to higher adiposity. Intriguingly, the authors also reported that adipocytes of the obese AT have an impaired oxygen uptake and consumption, which is paralleled by mitochondrial dysfunction and reduced AT capillarisation [80]. Although this finding has been recently disputed by Lee et al., who elegantly demonstrated an increased but futile oxygen consumption of adipocytes due to adenosine nucleotide translocase-2 (ANT2)-mediated uncoupled respiration, thereby inducing a relative state of cellular hypoxia [72]. Nevertheless, these findings highlight that oxygen flux within the adipocytes also regulates tissue homoeostasis. Indeed, exposing adipocytes to a hypoxic condition has been shown to alter the adipokine profile and insulin signalling [81,82], but whether the *in vitro* oxygen deprivation reflects a physiological level of hypoxia is of concern. Furthermore, AT PO_2_ is inversely associated with genes involving ECM remodelling and inflammatory markers, including interleukin-6 (*IL6*) and tumour necrosis factor (*TNF*), while expression of *VEGF* is positively related to AT oxygenation [77]. Additionally, expression of CD248 in response to hypoxia is positively associated with pathways enriched in blood vessel development, ECM organisation and inflammatory cascades [83]. This is further demonstrated that AT-specific CD248 knockout attenuates hypoxic response and HFD-induced inflammation and fibrosis, accompanied by improvements in adiponectin synthesis and glucose homoeostasis [83]. Collectively, these findings underlie the interdependence between hypoxia and defective angiogenesis in which they work in synergy to favour a pro-fibrotic and pro-inflammatory microenvironment. Ultimately, a pathologic ECM remodelling is coupled with AT immune activation, where inflammation, fibrosis and impaired angiogenesis interact reciprocally to provoke obesity-induced AT dysfunction [2].

## Reprogramming by immune modulation

Unlike acute inflammation defined by heat, redness, pain and swelling, the diseased state of obesity is underlaid by chronic sterile inflammation associated with metabolic pathologies or ‘meta-inflammation’ [84], where obesity stimulates a re-composition of the immune architecture within the AT [85]. Importantly, AT inflammation is the dominant contributor to systemic inflammation, thus creating a malignant environment that favours chronic diseases [86,87]. The metabolic and immune regulations of AT are often intertwined, in that an increased and sustained insulin signalling in chronic overnutrition pushes the adipocyte storage capacity beyond the limit, leading to cellular stress and dysfunctional lipid homoeostasis. The failure of buffering excessive lipids by the insulin-resistant AT, coupled with gut dysbiosis, contributes to rising levels of circulating free fatty acids (FFAs) and lipopolysaccharides, which in turn induce nuclear factor (NF)-kB signalling mediated by pattern recognition receptors (PRRs), specifically toll-like receptors on ATMs and adipocytes [88,89]. Induction of PPRs and NF-kB signalling primes NLR family pyrin domain containing - (NLRP3) inflammasome activation, which has a substantial role in AT dysfunction and meta-inflammation [90].

Local expansion and infiltration of ATM are among the phenotypic signatures of an inflamed AT. Specifically, macrophages comprise 4–10% of AT cellularity in lean state, but can increase up to 50% during obesity. Moreover, they are the dominant producers of pro-inflammatory adipokines and hence, major drivers of AT pro-inflammatory reprogramming [91–93]. Such expansion of ATM population is also accompanied by a phenotypic switch from an immune-modulatory M2 state to an immune-active M1 state positive for CD11c, hence leading to up-regulation of pro-inflammatory signalling but the suppressed resolution [94]. Note that conventional dendritic cells (cDCs) in AT also express CD11c but has a tolerogenic phenotype supporting an anti-inflammatory VAT microenvironment [95], hence the definitive roles of DCs and M1 ATMs require careful interpretations. Obesity-induced phenotypic shift and population accrual are also observed in other immune cell types, including a predominance of CD8^+^ T cells, especially those expressing metallothionein, and Th1 cells over T regulatory cells and Th2 cells [96,97]. These cells in turn aggravate IR and macrophage infiltration [98,99]. Moreover, ablating and restoring the pro- and anti-inflammatory cell lineages respectively have been demonstrated to relieve HFD-induced inflammation and restore insulin sensitivity [100,101].

Recent advances in single-cell RNA sequencing (scRNA-seq) have offered insights into novel AT immune populations, including the previously uncharacterised group 3-innate lymphoid cells (ILC3s), type 2B cDCs and inflammatory macrophages (IMs), and their extensive interactomes, which underpin the AT inflammatory milieu [102]. Specifically, obesity induces expansion and reprogramming of AT-resident cDCs and ILC3s and migratory monocytes, which, under the influence of pro-inflammatory adipokine and hypoxia signalling, preferentially differentiate into IMs and lipid-associated macrophages (LAMs) to avidly produce IL-1B and TNF in the obese fat [102]. Moreover, an enrichment of ILC1s that predominantly produce interferon-γ (IFNγ) in the omental fat is associated with human diabesity, and injection of AT ILC1s leads to VAT fibrosis through promoting M1-like ATM expansion and macrophage TGFβ-Smad3 signalling [103]. Although our understanding regarding AT immunity is largely attributed by studies delineating single cell lineage, it is important to recognise that AT is an active immune reservoir where different immune cell populations work synergistically and additively while interacting with surrounding adipocytes, ECM components and stromal cells to orchestrate the metabolic–immune homoeostasis [104–106]. Hence the extensive cellular cross-talk confers AT immune cells with the remarkable ability to be reprogrammed in response to surrounding niche and nutritional status. Such a dynamic cellular landscape has been elaborated by studies identifying heterogeneous immune populations performing distinct AT-specific physiological and homoeostatic roles [107,108], whose functionalities shift during obesity progression [109]. For example, in contrast with the conventional invariant natural killer T (iNKT) cells that are mainly involved in pro-inflammatory and carcinogenic responses, AT-resident iNKT cells, under the influence of FFA-enriched niche, acquire a unique phenotype that lacks PLZF expression but is positive for T-bet, GATA3 and E4NP4. These PLZF^low^ iNKT cells have major regulatory roles in lean mice through promoting ATM M2 polarisation and T-regulatory cell expansion mediated by the adipo-cytokines IL10 and IL2, conferring protection against DIO and adipose inflammation [110,111]. Subsequently, by using scRNA-seq, two subsets of AT iNKT cells distinguished by expression of NK1.1 are identified in the obese AT, where NK1.1^−^ and NK1.1^+^ cells act on the opposites of a spectrum by producing IL10 and IFNγ respectively [112]. Paradoxically, during the lean state, IFNγ produced by NK1.1+ cells perform an anti-inflammatory role by signalling NK cells to protect against pathologic ATM expansion [112]. Similarly, ILC3s, which is a novel cell population only found in human AT, have homoeostatic roles in lean fat through interacting with preadipocytes and adipose ECs but amplify AT inflammation through expressing IL6 [102]. Elucidating the mechanisms behind the fine-tuning of AT immune cell functions by different nutritional status will shed light on new therapies to reprogramme the AT microenvironment.

There is emerging evidence regarding the non-canonical functions of AT immune cells beyond immune-modulation, including angiogenesis and browning/beiging [105,113–115]. In addition to their anti-inflammatory properties, the M2-like ATMs are activated during a cold challenge to secrete the signalling protein SLIT3, which promotes the AT sympathetic nerve fibres to release norepinephrine and to nudge the white adipocytes into thermogenic adipocytes [116]. Similarly, tissue-resident γδ T cells, which are required for T regulatory cell accumulation, have crucial involvement in adaptive thermogenesis and brown AT innervation [58,117]. Conversely, sympathetic neuron-associated macrophages (*Cx3cr1^+^* SAMs) that are recruited to the nerve bundles of WAT during obesity can degrade norepinephrine, which is otherwise crucial for lipolysis and thermogenesis [113]. However, the *Cx3cr1^+^* macrophages are reportedly IL27 producers, where administration of IL27 protects against IR and obesity through its direct actions on *Ucp1^high^* adipocytes [118]. Such functional discrepancies of ATMs add another layer of complexity to the heterogeneity of AT immune populations. Future research aiming to leverage this cellular heterogeneity could offer new tools in favouring an adaptive AT immuno-reprogramming.

Indeed, ATM accumulation and CLS formation are hallmarks of AT dysfunction, the recruitment of phagocytic ATMs forming CLS can also be considered an adaptive response by AT to eliminate necrotic debris and to prevent loss of homoeostasis. In contrast with the scRNA-seq study reporting the pro-inflammatory role of LAMs, these CLS-containing LAMs expressing CD9 and Trem-2 also exert beneficial influences over AT remodelling during obesity by counteracting hypertrophy and preventing adipocyte death, while LAM ablation leads to enhanced weight gain and metabolic impairments [119]. Nevertheless, significant increases in LAMs and ATMs undergoing lipid-handling reprogramming have been identified using single-nucleus RNA sequencing in the VAT of obese mice [120], but it is yet to be defined whether such a population shift is a depot-dependent adaptation against the lipid burden or is at the expense of AT functionality. Moreover, abolishing the AT inflammatory signalling has been shown to dampen angiogenesis and inhibit AT browning, which is accompanied by fibrosis and IR, despite a reduced weight gain and an increased M2 ATM polarisation upon HFD [121]. Fasting and calorie restriction (CR), which are potential strategies against obesity comorbidities, are also found to be associated with an enrichment of ATM population during the intervention, specifically phagocytic macrophages, whose roles may include activation of non-canonical lipolysis and clearance of cell debris [122–124]. Again, these results underline the pleiotropic and dynamic roles of AT inflammation across different biological scenarios, and further understanding regarding the temporal control of the AT immune landscape during disease progression is needed.

## Reprogramming by cell cycle arrest and senescence

In addition to inducing pathologic AT reprogramming, obesity *per se* is also a major driver of biological ageing and ageing-associated diseases [125]. In fact, the Mouse Ageing Cell Atlas has shown that ageing is invariably associated with increased cellular and genetic signatures of senescence and inflammation across the 23 tissues/organs, including multiple depots of AT, where ageing-associated senescence also seems to arise the earliest [126–128]. Specifically, an abnormal accumulation of cellular senescence is a common denominator and contributor of ageing- and obesity-associated metabolic pathologies, while removal of senescence burden mitigates HFD-induced metabolic dysfunction, rejuvenate physical resilience, and extend healthy lifespan [129,130]. How do cells become senescent in the first place?

Mechanistically, cell cycle arrest instigated by DNA damage response (DDR) is coupled with and exacerbated by aberrant immune–metabolic responses in obesity, characterised by meta-inflammation, mitochondrial dysfunction and oxidative stress [131,132]. In particular, dysregulated ROS production is integrative to the initiation and maintenance of DDR and cellular senescence through p38 mitogen-activated protein kinase (MAPK) and p21-MAPK14-TGFβ1 signalling pathways [133,134]. Once become senescent, these cells adopt senescence-associated secretory phenotypes (SASPs), which foster inflammatory and fibrotic signalling, as well as propagating senescence in surrounding cells [135]. Note that the pro-inflammatory SASPs are also implicated in immunosurveillance and clearance of precancerous cells, and functionalities of SASPs are shifted to be anti-inflammatory and anti-fibrotic under appropriate spatial and temporal influences [136]. Moreover, the recent proteomic study elucidates the heterogeneous phenotypes of SASPs, which are largely driven by the initial senescence stimuli and the surrounding tissue microenvironment [137]. Hence, defining the AT-specific inducers of cellular senescence and the signature of SASPs are fundamental for understanding their roles in obesity and its associated diseases.

Cellular senescence of AT is one of the pathologic hallmarks of chronological ageing and obesity. Indeed, an obese AT is similar to an ‘ageing’ one, both of which are characterised by a hypertrophic morphology and a pro-inflammatory and pro-fibrotic secretome [138]. In the rodent model of diabesity, increased senescence markers, including p53 and γ-H2AX, of AT are coupled with an imbalance between pro- and anti-inflammatory adipokines and an increased number of pro-inflammatory ATMs [139]. Specifically, the visceral depot seems to be more susceptible to be senescent and expresses higher levels of *p53, p21* and *p16* during DIO or ageing [140,141]. More importantly, the ‘ageing’ AT can play a dominant role in instigating metabolic diseases since removal or implantation of the senescent AT rescues or exacerbates HFD-induced pre-diabetes respectively. Mice with AT-specific deficiency of *p53* also exhibited better maintenance of immune homoeostasis and adipocyte insulin sensitivity when fed a Westernised diet, underscoring the senescence machinery in the pathologic reprogramming [139]. More direct evidence comes from the observation that VAT-specific clearance of cells expressing *p21* (*p21*^high^), which attenuates AT senescence-associated β-galactosidase (SABG) activity, is sufficient to protect against DIO-induced systemic IR without affecting pancreatic β-cell function, indicating a causal role of the senescent AT in the T2D pathogenesis [142]. Interestingly, the obesity-associated AT senescence also induces anxiety-like behaviours and hypothalamic senescence, probably mediated by the actions of SASPs [143]. Given that the arcuate nucleus of the hypothalamus is the master regulator of appetite and hence the risk of weight gain [144], hypothalamic senescence in obesity may have spillover damage on appetite regulation and facilitates overeating, establishing a vicious cycle of weight gain through the AT–brain cross-talk. But such a hypothesis warrants further investigation.

The associations between AT cellular senescence and disease risk profile are also consistently shown in human studies, where an increased senescence burden in AT has been observed in patients with obesity, pre-diabetes/T2D and pre-frailty [13,145–147]. Importantly, subcutaneous adipocytes in participants with obesity and hyperinsulinemia, which are thought to be post-mitotic and hence less prone to be senescent, are capable of re-entering the cell cycle, evidenced by the expression of cell cycle markers cyclin A2/D1, and thereby proceeding towards senescence. Specifically, such cell cycle reprogramming is characterised by an absence of mitosis and increased nuclear content, known as endoreplication, which is otherwise advantageous for cells during regeneration and compensatory hypertrophy. However, under chronic insulin signalling, these hypertrophic adipocytes fail to be functionally adaptative and acquire senescence phenotypes that pave the way for AT inflammation and metabolic dysfunction. Moreover, in contrast with the animal findings where senescence is often observed in the visceral depot, the authors reported that cellular senescence in humans has a minor role in reshaping the omental AT compared with the subcutaneous depot [13]. It is illuminating to observe how insulin may serve more of a mitotic stimulus than an anabolic signal during metabolic dysfunction, but questions remain on what triggers the adipocytes to respond to insulin by re-entering the cell cycle in a depot-specific manner and whether the downstream targets and signalling cascades of insulin have altered in the senescent cells. Although most evidence has pointed that eliminating the senescence burden is a promising strategy to improve systemic metabolic profile, it is also likely that the pre-senescence signalling helps maximise the storage capacity of the subcutaneous depot, hence alleviating the hypertrophic burden on VAT and preventing systemic lipotoxicity and ectopic fat deposition [148]. Also, adipocytes without growth arrest are more hypertrophied and susceptible to necrotic death. Therefore, defining the dynamics of adaptive and pathologic senescence in obesity may be of relevance in the therapeutical manipulation of AT, especially for the ageing populations.

In addition to adipocytes, senescence is also implicated in AT immune cells, preadipocytes/adipocyte progenitors and ECs. Indeed, by leveraging transcriptomics at the single-cell level, the accumulation of senescent *p21*^high^ cells in DIO mice are shown to be mostly derived from preadipocytes, ATM and ECs, whose genes are enriched in inflammatory pathways but depleted for angiogenesis [142]. A healthy expansion of AT requires appropriate recruitment of adipocyte progenitors with a proliferative potential to favour hyperplasia over hypertrophy, which is linked to IR and AT mal-reprogramming. However, the reduced adipogenic potential observed in ageing or obesity is not due to a diminished pool of progenitors, but instead, to an increased senescence burden on adipocyte precursors [146]. Importantly, a single injection of senescent preadipocytes is sufficient to induce physical deterioration lasting up to 6 months in young healthy mice [129]. Moreover, these senescent progenitors, which are positive for SABG, γ-H2AX and cyclin-dependent kinase (CDK) inhibitors, have been shown to facilitate AT homing of macrophages and repress the expression of adipogenic regulators peroxisome proliferator-activated receptor-γ (*Pparg)* and CCAAT/enhancer-binding protein-α (*C/ebpa)* in non-senescent progenitors through the actions of SASPs. Conversely, ablation of senescent progenitors through targeting the p38MAPK-p16^Ink4a^ pathway or JAK signalling restores adipogenesis, inflammageing and enhances cold-induced beiging of WAT due to increased recruitment of beige precursors [149,150]. Interestingly, elimination of cellular senescence is also associated with preserved fat mass during ageing, where age-dependent lipodystrophy is otherwise implicated in metabolic and physical impairments in the old mice [151,152]. Whether the senescent adipocyte precursors can be a potential target in congenital lipodystrophy, a rare genetic disorder associated with a near-absence of SAT and systemic metabolic dysfunction [153], remains to be clarified. Furthermore, cellular senescence of AT mesenchymal stem cells in the elderly or those with obesity is also associated with a dampened cellular repair and angiogenic response and a reduced expression of Sirtuin-1 (SIRT1), a positive regulator of anti-inflammatory response and AT beiging/browning [145,154,155].

As discussed earlier, a sufficient vascularisation through angiogenesis is critical for supporting adaptive reprogramming of AT, where the angiogenic response is dependent on the extensive cross-talk between adipocytes and adipose tissue endothelial cells (ATECs) [156]. To note, individuals with obesity suffer from ATEC senescence characterised by γ-H2AX expression, and such senescent signature is found more prominent in VAT than SAT, potentially due to differences in depot micro-environment. Importantly, the senescent ECs in the obese VAT also exhibit an up-regulated gene expressions of monocyte chemoattractant protein-1 (*MCP1*),* IL8* and *VEGFR2* that encourage VAT angiogenesis and inflammation [69]. Therefore, cellular senescence may be one of the mechanisms explaining the discrepancies in the literature reporting the roles of angiogenesis in obesity, where a senescence-driven angiogenic response reflects a pathological reprogramming of AT vasculature. Intriguingly, both senescence and VEGF signalling reprogramme ECs towards glycolytic metabolism, suggestive of a similar metabolic response to pro-senescence and pro-angiogenic signals [157]. Hence, how senescent ATECs mediate dysfunctional angiogenesis in an obese AT is of future research interest.

ATECs are also involved in AT lipid transport and homoeostasis through the expression of fatty acid transporters and CD36 and secretion of PPARγ ligands, where activation of PPARγ is shown to promote AT angiogenesis and adipogenic differentiation of pre-adipocytes [158,159]. In fact, it has been revealed that senescent ATEC is associated with blunted lipid uptake in response to PPARγ, which may precipitate higher circulating FFAs and ectopic lipid deposition. Instead, these senescent ATEC have higher nuclear translocation of NF-kB and Forkhead box protein O1 (FOXO1), demonstrating that the anti-inflammatory and insulin-sensitising effects of PPARγ do not translate in the context of cellular senescence, which may deserve consideration in pharmaceutical development [160]. The pathologic influence of senescent ECs is also underpinned by a recent study, which showed that global EC senescence induces adipocyte oxidative stress and systemic IR through down-regulating adipocyte insulin signalling and propagating WAT senescence. Interestingly, the WAT is the most susceptible to the deleterious impact of systemic EC senescence compared with other organs [161]. Future efforts are required to define the physiological role of ATEC senescence in obesity complications and the cross-talk between ATEC and other metabolic organs.

## Reprogramming by circadian rhythm

Several lines of evidence supporting the positive associations between chronic shift work or (social) jet lag and incidence of metabolic diseases have led to an increasing appreciation of the contribution of circadian misalignment in the pathogenesis of obesity and the influences of clock genes in AT homoeostasis [162–164]. The circadian clock system is a multi-oscillatory network comprising a master clock in the suprachiasmatic nuclei (SCN) [165], and peripheral clocks running autonomously in all other body tissues, which can be entrained by SCN through electrical, endocrine and metabolic pathways, although the precise mechanisms are yet to be defined [166].

The molecular machinery of the circadian system has been elegantly reviewed here [167,168]. Briefly, our internal timekeeper is based on transcription–translation feedback loops consisting of circadian locomotor output cycles kaput (CLOCK)–brain and muscle ARNT-like 1 (BMAL1) heterodimers and their target clock genes Period (*PER 1-3)*, Cryptochrome (*CRY 1-2*),* REV-ERB* and retinoic acid-related orphan receptor (*ROR*). Accumulation of these clock proteins then serve as repressors of CLOCK-BMAL1 and hence their own transcriptions, generating oscillations in gene expressions and creating an antiphase relationship between *BMAL1* and *PER/CRY* expressions. The circadian oscillations of CLOCK–BMAL1 heterodimer then regulate the transcription rhythmicity of clock-controlled genes containing E-box sequences. Despite operating on a common feedback mechanism, these core clock machinery regulate crucial but diverse pathways in a tissue-specific manner, where there are very few overlapping clock gene outputs between different organs [169,170].

One of the pioneering studies investigating the association between obesity and circadian dysfunction comes from the observation of *Clock* mutant mice, where global *Clock* mutation leads to hyperphagia, obesity and metabolic pathologies independent of diet. Specifically, the weight gain of *Clock* mutant mice is largely attributed to visceral adiposity, which is accompanied by AT hypertrophy [171]. By contrast, administration of Rev-Erbα agonist reduces adiposity and mitigates systemic IR and inflammation in DIO mice [251]. Furthermore, AT-specific deletion of *Bmal1* or *Cry* also promotes significant weight gain and higher adiposity, which is accompanied by the formation of hypertrophic adipocytes and CLS during HFD [172,173], implying that dysfunctional clock operation is possibly the pathogenic driver and accelerator of metabolic abnormalities. Indeed, genetically obese (*ob/ob* or KK-A^y^) or DIO mice have reportedly altered or dampened oscillations of clock genes in VAT, where the abnormal AT rhythmicity precedes the development of morbid obesity and metabolic dysfunctions [174,175]. Although there are inconsistencies regarding the influences of obesity on human AT clock machinery, the number of rhythmic genes in SAT is shown to be reduced by more than fourfold in patients with obesity and T2D compared with lean individuals, which is coupled with an abnormal oscillatory transcriptome implicated in AT metabolic and immune dysfunction [176,177].

In fact, genes involved in AT homoeostasis and functionality, including fatty acid metabolism, adipogenesis and thermogenesis, also demonstrate robust circadian oscillations driven by the core clock machineries, underscoring the extensive interconnections among metabolic, immune and circadian pathways in AT [178–180]. In WAT, expression of transcripts encoding the lipogenic enzymes (elongation of long-chain fatty acids family member 6 (*Elovl6*) and stearoyl-CoA desaturase enzyme 1 (*Scd1*)) and lipolytic enzymes (hormone-sensitive lipase (*Lipe*) and adipose triglyceride lipase (*Pnpla2*)) are under the direct influences of core clock components BMAL1 and CLOCK, which confer the diurnal variability in lipid turnover and serum lipid profile [181,182]. The core clock component Rev-Erbα also functions as a potent transcription regulator in WAT and directly down-regulates transcriptions of lipoprotein lipase [183], fibroblast growth factor-21 (FGF21) cofactor [184], and enzymes involved in triglyceride synthesis [185]. A similar suppression on adipogenesis and fatty acid metabolism by Per2 through its direct interaction with PPARγ in WAT has also been suggested [186].

Additionally, NF-κB, the master regulator of AT inflammatory cascade, represses BMAL1/CLOCK transcriptional activity, thus causing circadian clock dysfunction in response to inflammation [187]. A recent study further suggests that chromatin binding by BMAL1 has profound influences over genes involved in AT reprogramming processes, including inflammation, ECM remodelling and hypoxia response [175]. Specifically, there is a repositioning of BMAL1 genome-wide occupancy in the obese VAT, such that an up-regulated production of pro-inflammatory adipokines is observed. These observations highlight the interconnections between circadian and immune reprogramming of AT, which is also evidenced by the close proximity of BMAL1 to the NF-κB consensus motif [175].

Similar to its white counterpart, the brown AT is profoundly influenced by circadian reprogramming, and ∼8% of the protein-coding genes in brown AT are clock-controlled [188]. Specifically, uptake of fatty acids and glucose by brown AT demonstrates strong diurnal rhythmicity, and up to 12-fold differences between highest and lowest FA uptake within a day–night cycle are found [189,190]. These variations coincide with the oscillating expressions of lipoprotein lipase, angiopoietin-like 4, glucose transporter-4, and *UCP1*, which are also in antiphase with *REV-ERBA* and *BMAL1* [191,192]. Mechanistically, BMAL1 has a suppressive effect on brown adipogenesis and brown adipocyte commitment, which is mediated by enhanced activity of TGFβ relative to the bone morphogenetic protein signalling [193]. Surprisingly, brown AT-specific deletion of *Bmal1* significantly down-regulates tissue metabolism and thermogenesis, which is accompanied by greater weight gain during HFD despite an up-regulation in *Ucp1* [194]. Further investigation unravelling the impact of clock machinery in regulating sympathetic innervation and UCP1-independent thermogenesis of brown AT is needed. Similarly, Per2, the transcriptional suppressor of BMAL1, is found to be indispensable for UCP1-dependent cold-induced thermogenesis, where Per2 mutation leads to cold intolerance and defected brown AT metabolism [195]. Collectively, an intact and optimised circadian system is critical for adaptive reprogramming of both white and brown depots, and an improved understanding of circadian regulation of AT could yield novel obesity therapeutics. Intriguingly, despite enhancing systemic circadian rhythms in DIO mice through small molecules has shown to alleviate metabolic disease burden [196], the benefit of specifically targeting AT circadian rhythmicity in obesity is unexplored.

## AT reprogramming as therapeutic tools

**Figure 2:**
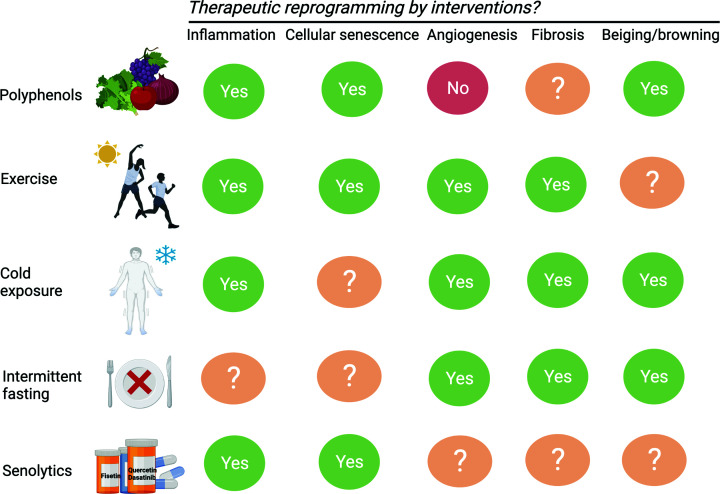
Interventions favouring adaptive reprogramming of AT Nutritional, pharmacological and lifestyle interventions demonstrating efficacy in rectifying metabolic diseases through modulating the phathological reprogramming processes. The mechanistic targets of each potential intervention are illustrated.

The mainstream anti-obesity therapeutics have been centred around manipulation of appetite regulation and hence the food intake component in the energy balance equation. Indeed, such approaches have recently offered significant breakthroughs in tackling the obesity crisis, especially in the case of glucagon-like peptide-1 analogues [197]. On the other hand, sympathomimetics, such as phentermine, are prescribed to induce thermogenesis and lipolysis through direct stimulation of the β-adrenergic receptors [198]. However, the prevailing unwanted effects of anti-obesity medications, including cardiovascular events and gastrointestinal discomfort, render intolerability and safety concerns [199]. Advances in recent obesity therapeutics have been articulated by Muller et al. [200]. Here, we will broadly summarise the therapeutics that modulate AT reprogramming, thus targeting the root cause of metabolic dysfunction, in addition to their proposed benefits on weight loss or maintenance (Figure 2).

Involvement of AT senescence burden in obesity has made senolytics, which are mostly naturally occurring polyphenols targeting senescent cells, effective in rescuing AT dysfunction and its associated complications. Importantly, senolytics, especially when used in combination, target multiple cell damage and pro-survival pathways, including inhibition of HIF1A, PI3K (phosphatidylinositol 3-kinase)/Akt (protein kinase B)/mTOR (mammalian target of rapamycin) and B-cell lymphoma 2 (Bcl2) anti-apoptotic signalling, hence inducing apoptosis in senescent cells without off-target effects [201]. The combination of quercetin and dasatinib restores insulin sensitivity and subcutaneous adipogenesis in obese mice, accompanied with suppressed SASPs and reduced monocyte infiltrate in AT, although the resident ATM populations are unaltered by senolytics [129,130]. By leveraging mass cytometry, the senescent adipocyte progenitors, CD4^+^/CD8^+^ T cells, NK cells and ATECs in AT, but not ATMs expressing *p16*, are identified as the cellular targets of the senolytic polyphenol [202]. In fact, expressions of *p16* and SABG activity are transiently elevated in macrophages when reprogramming towards an M2-like phenotype [203], which again, demonstrates the high specificity of senolytics in targeting *bona fide* senescence. In addition to their senotherapeutic actions, many of the polyphenols, such as epigallocatechin gallate, curcumin and resveratrol derived from green tea, turmeric and grapes respectively also demonstrate potent anti-inflammatory and insulin-sensitising properties as well as ameliorating the pathologic reprogramming of AT and extending life span [204–206]. These are achieved at least partially through influencing the gut microbiota and its metabolites, which are implicated in reshaping the AT during metabolic stress [207–209]. Note that resveratrol has shown to decrease expression of angiotensin-converting enzyme-2 (*ACE2*), which is the cellular entry point of SARS-CoV-2, in human SAT, but the clinical implications of resveratrol on COVID-19 are yet to be examined [210]. Moreover, capsaicin and capsinoids derived from chilli peppers have been shown to protect against DIO and dyslipidaemia through inducing expression of beiging factors in WAT and enhancing the sympathetic tone and vascularity of brown AT [211–213]. Importantly, scRNA-seq study has shown that adipocyte progenitors expressing transient receptor potential vanilloid 1 (*Trpv1*), the molecular target of capsaicin, are predominantly responsible for cold-induced thermogenesis and beiging through differentiating into brown and beige adipocytes [214], suggesting the mechanistic role of capsaicin as a browning/beiging agent. Benefits of these natural compounds are also supported by human dietary interventions reporting improvements in metabolic profile and body weight control by adopting a plant-based Mediterranean dietary patten [215,216].

In addition to pharmaceutical approaches, exercise has shown to alleviate the senescent and inflammatory signature of WAT [140,217,218]. Interestingly, transplantation of SAT taken from exercised mice is sufficient to benefit systemic glucose homoeostasis in sedentary mice [219,220]. Moreover, improvements in AT vasculature and glucose homoeostasis are also evident in insulin-resistant individuals [221], implying an adaptive reprogramming of AT by exercise. Paradoxically, despite an increased body temperature, exercise leads to enhanced sympathetic activity and beiging/browning of murine AT as evidenced by the up-regulation of key marker genes and the presence of multilocular cells in the subcutaneous depot [222,223]. Although the effect of habitual exercise on human brown AT activity is controversial [224,225], which can be confounded by concurrent cold challenges during winter swimming, the beneficial adaptations of a ‘trained’ AT can justify exercise as an adjunct anti-obesity therapy. Specifically, in addition to inducing beiging/browning, cold exposure modulates monocyte activation and angiogenesis, and importantly, alleviates the inflammatory and fibrotic phenotype of AT, where recruitment of the PRDM16 (PR domain containing 16)–GTF2IRD1 (general transcription factor II-I repeat domain-containing protein 1) complex by cold exposure suppresses pro-inflammatory gene expressions and pro-fibrotic signalling in the adipocytes [226–228]. Nevertheless, whether the reduction in pro-inflammatory transcripts is attributed to cold-induced beiging/browning or cold exposure *per se* is unclear. The anti-inflammatory effect of cold exposure merits human investigations.

Daily CR is among the lifestyle modifications showing efficacy in delaying or preventing the onset of obesity and its comorbidities [3,229]. Yet, CR *per se* has not shown to be superior to prolonged intermittent fasting (IF) [230]. In fact, it is the fasting component that underlies the benefit of CR, where fasting without food reduction recapitulates the metabolic adaptations to CR [231]. Indeed, IF has been shown to benefit metabolic profile and promote adaptive reprogramming of AT, including up-regulation of mitochondrial metabolism and *Ucp1* expression and improvements in pro-fibrotic and pro-inflammatory burden [231–234]. Interestingly, markers of M1-like ATM are elevated in the SAT of women with overweight or obesity subject to IF [123], which may imply AT inflammation as a prerequisite or a priming signal for beneficial adaptations. Future trials with longer intervention periods could disentangle the differential roles of AT immuno-reprogramming during nutritional challenges. Specifically, time-restricted feeding (TRF), which implements a daily feeding period of ≤12 h and is designed for synchronising food intake with diurnal rhythms in metabolism, has shown to reverse DIO-induced complications and alleviate the burden of metabolic syndrome in individuals with overweight or obesity, despite a lack of dietary restriction and drastic weight loss [235–237]. In fact, a 10-h TRF significantly induces adipocyte VEGF expression and AT angiogenesis, which is accompanied by resistance to obesity and AT inflammation upon HFD. Interestingly, such fasting-induced reprogramming is abolished by deletion of hepatic FGF21 signalling, underpinning the liver–AT cross-talk in regulating metabolic homoeostasis [238]. Given that circadian misalignment dictates and accelerates AT pathologic reprogramming [239,240], further efforts are needed to confirm the potential of TRF as part of the toolkit for therapeutically reprogramming AT in humans. Additionally, it remains unclear whether one’s circadian pattern or chronotype influences the physiological responses of AT during TRF, and whether the misaligned AT clocks underlies the discrepancies in risks of obesity observed between the ‘morning larks’ and the ‘night owls’ [241–243]. Currently, bariatric surgery remains the ‘gold standard’ for treating morbid obesity with a high success rate that cannot be outrun by lifestyle or pharmaceutical interventions alone [200]. Further trials aiming to investigate the synergy of combining interventions that can therapeutically reprogramme AT and target appetite regulation could offer new avenues for non-invasive metabolic risk management.

## Implications of AT remodelling in COVID-19

In addition to managing chronic diseases, therapeutic reprogramming of AT may have wider implications on the current COVID-19 pandemic, considering that the AT has shown to be an infection reservoir of the SARS-CoV-2 and potentiates the systemic ‘cytokine storm’ of COVID-19 by adopting a pro-inflammatory phenotype upon viral infection [244]. Notably, infection of AT by SARS-CoV-2 has been found in male patients with overweight or obesity, and such infection is associated with adipose dysfunction characterised by reduced adiponectin and adipsin production, contributing to systemic IR and hyperglycaemia and impaired *de novo* lipogenesis [245,246]. Although the relationship between obesity and adipose expression of *ACE2* is debatable [246,247], the insulin-resistant and obese AT is associated with T-cell exhaustion due to programmed death-ligand 1 (PD-L1) overexpression [248], altered methylation of *ACE2* [249]. In addition, there is an up-regulated expression of glucose-related protein-78 (GRP78) in the obese AT, which acts as a co-receptor to facilitate the interaction between SARS-CoV-2 and ACE2 [250]. Collectively, these studies suggest a greater infection susceptibility of the ‘sick’ fat. Interestingly, dietary and lifestyle interventions, including exercise and cold exposure, have been shown to reduce adipose expressions of *GRP78* and neuropilin-1 (*NRP1*), which is a known SARS-CoV-2 entry factor [249–251]. However, it is probably that SARS-CoV-2 may also manoeuvre the AT niche and exploit it as a pathogenic hub favouring virus replication and transmission, and such viral reprogramming of AT needs to be resolved. Given the dominant role of AT in regulating metabolic homoeostasis and the latter one is a strong predictor of infection outcomes [252], the benefits of ‘educating’ the AT as an adjunct to COVID-19 prevention and vaccination warrants longitudinal examinations.

## Conclusion

Our knowledge of obesity as a metabolic disease has been evolved from a central focus on appetite and energy homoeostasis to a growing appreciation on peripheral influences by metabolic organs, specifically the pathologic reprogramming of the WAT. This is in turn, intricately linked to the central ‘lipostat’ [253] and systemic homoeostasis. Moreover, our ability to sequence at the single cell and single nucleus resolution and profile metabolites with an increasing level of sophistication has offered novel insights into the potential of targeting the dysfunctional AT as therapeutic alternatives. However, the multifaceted and dynamic nature of the AT reprogramming poses challenges in understanding the temporal course leading to the ‘sick AT’ during the onset and progression of obesity. Such challenges are further escalated by the heterogeneity and plasticity of AT cell populations, which are extraordinary in the sense that they can be readily reprogrammed by the tissue microenvironment and subsequently re-establish a new homoeostatic set-point. Such that, the AT can still retain its obesogenic memory after weight loss and HFD reversal, leading to an uncoupling of adiposity and metabolic dysfunction and facilitating weight rebound and cycling, which in turn, disrupts immuno-metabolic homeostasis [254–256]. Moreover, questions remain on the triggers leading to the ‘obese and sick’ AT and whether these events vary between individuals based on their baseline risk factors, chronotypes and the gut microbiome. Understanding the interindividual differences in the AT architecture and illuminating the evolving functional landscape of AT and its cell populations during obesity progression will help develop tailored management and promote better adaptations to interventions. Lastly, it is important to realise that these reprogramming processes alone cannot be definitively labelled as either ‘pathologic’ or ‘adaptive’; instead, the reprogramming potential of AT should be considered as an evolutionarily conserved mechanism accommodating environmental and metabolic fluctuations and a powerful anti-obesity tool to be leveraged.

## Data Availability

The data are available from the corresponding author upon reasonable request.

## Open Access

Open access for this article was enabled by the participation of Chinese University of Hong Kong in an all-inclusive *Read & Publish* agreement with Portland Press and the Biochemical Society under a transformative agreement with JULAC.

## Abbreviations

ACE2: angiotensin-converting enzyme-2
Akt: Protein kinase B
AT: adipose tissue
ATEC: adipose tissue endothelial cell
ATM: adipose tissue macrophage
Bcl2: B-cell lymphoma 2
BMAL: brain and muscle ARNT-like 1
cDC: conventional dendritic cell
CLOCK: circadian locomotor output cycles kaput
CLS: crown-like structure
CR: calorie restriction
CRY: cryptochrome
DDR: DNA damage response
EC: endothelial cell
ECM: extracellular matrix
Elovl6: elongation of long-chain fatty acids family member 6
FFA: free fatty acid
FGF21: fibroblast growth factor-21
GRP78: glucose-related protein-78
GTF2IRD1: general transcription factor II-I repeat domain-containing protein 1
HFD: high-fat diet
HIF1α: hypoxia-inducible factor-1α
IF: intermittent fasting
IFNγ: interferon-γ
IL: interleukin
ILC: innate lymphoid cell
iNKT: invariant natural killer cell
IR: insulin resistance
JNK: c-Jun N-terminal kinase
LAM: lipid-associated macrophage
Lipe: hormone-sensitive lipase
MAPK: mitogen-activated protein kinase
mTOR: mammalian target of rapamycin
NF-kB: nuclear factor κ-light-chain-enhancer of activated B cells
NLRP3: NLR family pyrin domain containing-3
PD-L1: programmed death-ligand 1
PER: period
PI3K: phosphatidylinositol 3-kinase
Pnpla2: adipose triglyceride lipase
PO_2_: partial pressure of oxygen
PPARγ: peroxisome proliferator-activated receptor-γ
PRR: pattern recognition receptor
PRDM16: PR domain containing 16
ROR: retinoic acid-related orphan receptor
SABG: senescence-associated β-galactosidase
SASP: senescence-associated secretory phenotype
SAT: subcutaneous adipose tissue
scRNA-seq: single-cell RNA sequencing
Scd1: stearoyl-CoA desaturase enzyme 1
T2D: type 2 diabetes
TGFβ: transforming growth factor-β
TNF: tumour necrosis factor
TRF: time-restricted feeding
UCP1: uncoupling protein-1
VAT: visceral adipose tissue
VEGF: vascular endothelial growth factor
VEGFR: vascular endothelial growth factor receptor
WAT: white adipose tissue
